# Comparative transcriptome analysis reveals candidate gene for flowering time QTL *HvHeading* in barley

**DOI:** 10.1186/s12870-025-06598-4

**Published:** 2025-06-20

**Authors:** Nazanin P. Afsharyan, Jens Léon, Ali Ahmad Naz, Agim Ballvora

**Affiliations:** 1 Institute of Crop Science and Resource Conservation, Chair of Plant Breeding, University of Bonn, 53115 Bonn, Germany; 2 Leibniz Centre for Agricultural Landscape Research (ZALF), 15374 Müncheberg, Germany; 3 Department of Plant Breeding, University of Applied Sciences, 49076 Osnabrück, Germany

**Keywords:** Barley, Comparative-transcriptomics, Flowering time, *HvSpt6*, MAGIC population, Epistatic loci, Elongation factor

## Abstract

**Background:**

Identifying genes regulating flowering time enhances understanding mechanisms that improve crop adaptation and productivity. This study aims to identify gene(s) underlying barley flowering time quantitative trait locus (QTL) “*HvHeading*”.

**Results:**

To investigate the reported delayed-flowering effect of QTL, we selected spring barley MAGIC DH lines with different alleles in *HvHeading* locus which carry the same alleles in epistatic loci. Phenotyping apex development revealed contrasting effects of two alleles of *HvHeading* locus. Combining recombination and differential gene expression analyses using RNA-sequencing for apex and leaf tissue pinpointed a 3.94 Mbs interval which carried 22 differently expressed genes. Initial analysis using Morex IBSC v2 reference genome suggested a transcription elongation factor *HvSpt6*, encoded by *HORVU1Hr1G067820*, as a possible candidate gene potentially involved in flowering time regulation. Full-length *HvSpt6* sequencing found two promoter mutations in the allele from delayed-flowering genotype, creating a binding site for *TEM1*, a transcription factor known for involvement in suppressing flowering time in Arabidopsis.

**Conclusions:**

The findings provided the first insights for flowering time regulation by *HvHeading* underlying gene. Though further functional studies are needed to conclusively identify the causal gene. This study showed that leveraging knowledge of epistatic loci to address phenotypic background effects, followed by RNA sequencing can be an effective approach for identifying genes in large regions of suppressed recombination in crops with complex genomes.

**Supplementary Information:**

The online version contains supplementary material available at 10.1186/s12870-025-06598-4.

## Background

Barley (*Hordeum vulgare* L.) is the fourth most cultivated cereal worldwide. It was one of the earliest crops to be domesticated since about 10,000 years ago and currently is used for food, feed and malting process [1]. Flowering at the optimal time is crucial for determining the final grain yield in barley. Timing of flowering in barley is orchestrated by interplay of genes within a complex genetic cascade and pinpointing the genes that fine-tune this trait can shed light on the mechanisms that improve yield [2].

To date, a few number of major genes are reported to have a substantial impact on flowering time [3–5]. The onset of flowering under long-day (LD) is modulated by the *PSEUDO-RESPONSE REGULATOR* gene *HvPRR37*, a major photoperiod response gene also referred to as *PHOTOPERIOD RESPONSE LOCUS1* (*Ppd-H1*) [6]. A natural mutation in the conserved CCT (*CONSTANS*, *CONSTANS-LIKE*, and *TOC1*) domain of *Ppd-H1* results in diminished responsiveness to LD [6]. The flowering is initiated when *Ppd-H1* promotes the expression of *Vrn-H3* gene, a homolog of *Arabidopsis thaliana FLOWERING LOCUS T* (*FT*) gene. Mutations in the first intron of the *Vrn-H3* gene, are causally linked with the dominant or recessive *Vrn-H3* alleles which respectively, promotes and delays flowering [7]. *Vrn-H1* gene, a member of the *APETALA1* family MADS-box transcription factors, is another major gene that positively regulates flowering time through its interaction with the downstream gene, *Vrn-H3* [8]. Deletions in a regulatory region of *Vrn-H1* in spring barley have been shown to be associated with a loss of vernalization requirement [9, 10].

Candidate gene identification in barley is hampered by its large and complex genome which includes extensive regions of reduced recombination, making the process for map-based approaches particularly time-consuming, costly and not as successful compared to cereals with smaller genome such as rice [11–13]. Publication of the first draft reference Morex genome [14], and its subsequent revisions [15–17] provided knowledge about barley physical gene space and the possibility to directly search positional candidate genes within the QTL interval, also known as the candidate gene approach [18–20]. Furthermore, next generation sequencing (NGS) approaches such as RNA-sequencing, currently offer tools to greatly fortify gene identification in barley providing information on gene expression, which in turn reduces the genome sequence complexity and the reliance on recombination [14, 15, 21–23]. Hence, the prioritization of candidate genes can be performed by leveraging sequence data, functional annotation, and the analysis of differential gene expression [18, 19, 24, 25]. RNA-sequencing can provide high precision and reproducibility for identifying transcript-regulated genes when data is collected from proper tissue and time-point, and analysis is carried on with sufficient sequencing depth [26]. This approach has been successfully utilized to identify genes in barley involved in tissue-specific albinism using near-isogenic lines (NILs) [27], to narrow down the candidate gene underlying a QTL for awn length using NILs [28], and to isolate six-rowed spike 3 (*VRS3*) gene using allelic mutants [23]. Validating the effect of a QTL and prioritizing the underlying candidate gene(s) requires evaluating the phenotypic effect of the QTL for the trait of interest. The accuracy of the approach is increased by eliminating the effects of the background genes to remove the noise that might obscure the effect of causative gene. It is traditionally conducted by a multi-step and multi-year process including developing near isogenic lines (NILs) that correspond to the region of interest and marker saturation of the region of interest in the progenies to narrow down the genetic region and prioritize the candidate genes based on the observed phenotype [21, 25, 29]. To facilitate the gene identification process, an alternative approach can be proposed by leveraging the information provided by epistasis mapping, to detect the loci that interact with the targeted region and explain a high portion of trait phenotypic variance. Then, this information can guide selecting genotypes with contrasting alleles at the locus of interest, which have the same allele at the respective epistatic locus, thereby removing the masking effect on the locus of interest and achieve a targeted phenotypic background effect elimination.

The multi-parent advanced generation intercross (MAGIC) strategy combines linkage-based design and multiple generations of recombinations to provide higher power and precision in QTL mapping [30, 31]. Previously, Afsharyan et al. [5] used a spring barley MAGIC population to map QTL and epistatic interactions that control flowering time in barley and detected “*HvHeading*”, a novel epistatic QTL on chromosome 1H with flowering-delaying effect. The flowering-delaying allele for *HvHeading* descended from parental line Danubia. This QTL was found to be involved in prominent epistatic interactions with *Ppd-H1*, *Vrn-H1,* and *Vrn-H3* loci which suggested that the underlying gene might have an important role in timing of flowering [5]. To trace the candidate genes underlying *HvHeading*, we selected and phenotyped contrasting MAGIC DH lines with different alleles in *HvHeading* locus that carry the same alleles in epistatic loci. RNA-Seq-based comparative transcriptomics of these lines led to the detection of the candidate gene *HvSpt6*, which encodes a transcription elongation factor. Furthermore, the allelic variation at the *HvSpt6* locus was analyzed. This study provides insights on flowering time regulation in barley which can be used to optimize this important breeding trait to improve crop yield.

## Methods

### Plant material

The DH lines were selected from a spring barley (*Hordeum vulgare* ssp. *vulgare*) MAGIC population that was used to map epistatic interactions for days to heading by Afsharyan et al. [5]. The population was constructed by inter-crossing eight barley genotypes, including one plant from each of seven landraces, Ackermanns Bavaria IPK No. HOR 100 (Ack. Bavaria), Ackermanns Danubia IPK No. BCC 1427 (Ack. Danubia), Criewener 403 IPK No. HOR 62, Heils Franken IPK No. BCC 1433, Heines Hanna IPK No. HOR 59, Pflugs Intensiv IPK No. BCC 1441, and Ragusa IPK No. BCC 1359, as well as the elite cultivar Barke, as detailed by Sannemann et al. [32]. Days to heading was measured when 3 cm of awns appeared for 50% of individuals from each DH line.

### Estimation of genetic recombinations in *HvHeading* QTL interval

The *HvHeading* QTL interval spanned from 67.13 cM (BOPA1_1670 − 369, 473.583Mbs) to 76.84 cM (SCRI_RS_181353, 488.815 Mbs) for 15.23 Mbs, containing 160 high-confidence genes with predicted functions (HC-G) [5]. The marker positions are reported based on Morex IBSC v2 reference genome [15], which was also utilized by the study preceding this research [5]. The list of SNPs in the QTL region according to barley 9k iSelect array [33], and the corresponding positions in reference genomes Morex IBSC v2 [15], and Morex v3 [17] were compared which revealed the distances between the majority of the markers are consistent across both reference genomes. The marker positions are presented in Supplementary Table 1. The position of SNPs among both reference genomes were compared using BARLEYMAP [34]. To determine the recombination frequency within the *HvHeading* QTL interval, this study utilized available genotyping data from 534 DH lines, consisting of 12 SNPs from barley 9k iSelect array [33], which cover a 9.98 cM interval in the *HvHeading* region [5]. Additionally, new data was generated from 279 DH lines using the most associated SNP marker BOPA1_1016 − 376, and three adjacent markers SCRI_RS_138527, BOPA1_6655 − 978, and SCRI_RS_181239 within the interval (Table S1). To generate the data, plants were sown in 96-cell growing trays (100 mL/cell) and the leaf tissue from seedlings was collected and immediately frozen in liquid nitrogen; then stored at -80 °C for DNA extraction. The DNA extraction was performed according to the protocol of Virginia Tech Small Grains Breeding (www.cropgenetics.cses.vt.edu*).* Developing of the KASPar markers based on the four described SNP markers targeting the QTL region, and genotyping procedures were conducted by TraitGenetics GmbH (Stadt Seeland OT, Gatersleben, Germany). In silico analysis was performed using the IPK barley BLAST server (https://webblast.ipk-gatersleben.de/barley_ibsc/) [35] and Ensembl Plants (http://plants.ensembl.org) [36].

### Selecting contrasting pairs of DH lines and phenotyping of main shoot apex development

Major genes involved in epistatic interactions with *HvHeading* including *Ppd-H1*, *Vrn-H1*, and *Vrn-H3* [5], were genotyped in parental lines using gene specific primers listed in Supplementary Table 2. The investigated genetic variations are nine SNPs for *Ppd-H1* gene described by Turner et al. [6], two SNPs for *Vrn-H3* described by Yan et al. [7], and a deletion in intron one of *Vrn-H1* described by Wang et al. [37], and were genotyped in founder-lines by Sanger sequencing. To determine the allele for each gene in DH lines, the parental haplotype for each respective loci was determined for DH lines based on available genotyping data from 534 DH lines [5]. To select contrasting pairs of DH lines for microscopy-based phenotyping of apex and early inflorescence, 534 DH lines were subjected to a rigorous selection process. Available SNP described by Afsharyan et al. [5] was used to divide the DH lines in two groups to separate the lines that carried the haplotype from parental line Danubia, the donor parent for flowering-delaying allele [5] in *HvHeading* interval, from the rest of genotypes. Then, two groups were compared to find DH lines that had the same allele for *Ppd-H1*, *Vrn-H3,* and *Vrn-H1.* The selected DH lines were further narrowed down to find pairs with the similarly originated haplotype blocks in the other seven flowering time QTL described by Afsharyan et al. [5]. The plants were sown in 96-cell growing trays (100 mL/cell). To equalize germination, moist seeds were kept in the dark at 4 °C for 3 days. Subsequent to germination, they were transferred to short-day (SD) condition (8 h, 22 °C day; 16 h, 18 °C night; PAR 270 µM m^− 2^ s^− 1^) in growth chamber. The plants were kept in SD condition for one week which was then switched to LD condition for 9 weeks (16 h, 22 °C day; 8 h, 18 °C night; PAR 270 µM m^− 2^ s^− 1^). For phenotyping main shoot apex (MSA) development, two separate experiments were conducted to validate the developmental stages. Three plants per genotype were dissected every two days from germination to heading. At each time-point, the developmental stage of MSA was determined and quantified according to the quantitative scale (Waddington stage; W) described by Waddington et al. [38]. In addition, data for days to heading were collected for 10 plants per genotype. Images of apices were obtained using Digital microscope KEYENCE model VHX-900 F (KEYENCE Corporation, Osaka, Japan). The development of apex and inflorescence of DH lines was compared by paired Student’s t-test. Broken-line regressions were calculated using the “SiZer” package in R software [39].

### Sample preparation for RNA-sequencing

DH lines were sampled for RNA-sequencing (RNA-seq). The growth condition was the same as the experiment for apex phenotyping. The apex and leaf tissues were harvested from the main shoots. Samples were collected three hours after lightening and three hours after darkening of climate chamber at 11 days, 19 days and 33 days after germination (DAG). Before each sampling, three plants per genotype were dissected, and the developmental stage was confirmed according to Waddington stage. For sampling the apex, the leaves surrounding the MSA were removed manually, and the apex was cut with a Paragon Nr.10 microsurgical disposable stab knife under a stereo microscope. From each tissue, a minimum of three pooled biological replicates were taken per time-point for each DH line. Each of the MSA samples collected during the vegetative phase W1.0 consisted of 30 pooled apices. During reproductive stages W1.5-2.0 and W3.5, pool of 15 and 11 shoot apices were collected, respectively. The leaf samples were harvested from a subset of 10 plants, from which apex tissue was collected. The harvested leaf tissue was restricted to the distal part of the leaf, at 2 to 4 cm before the leaf tip. On average, RNA-seq sample collection was completed within three hours each time. For each day the two apex pools collected in light and dark were mixed for each genotype, to mitigate the possibility of differences in gene expression in presence and absence of light. The harvested samples were immediately frozen in liquid nitrogen, then stored at -80 °C.

### RNA sequencing, quality control, and mapping

Total RNA extraction, library preparation, and sequencing was performed commercially at Novogene Co. Ltd. (HK, China). A set of 36 libraries corresponding to two genotypes, two tissue types, three time-points, and three biological replicates were constructed for the Illumina HiSeq PE150 platform, and 1,970,926,721 paired-end reads were generated. The sequencing data quality was verified using FastQC software (version 0.11.9; https://www.bioinformatics.babraham.ac.uk/projects/fastqc/*).* Read trimming was performed using the Trimmomatic program version 0.39 [40] to ensure the adaptors and short reads were removed using the following criteria: phred 33, leading and trailing 10, sliding window 30:15, headcrop 15, and a minimum read length of 70. Finally, 1,840,628,787 trimmed reads were used for down-stream analysis and aligned to the barley reference genome Morex IBSC v2 [15] (https://webblast.ipk-gatersleben.de/barley_ibsc/downloads/) using HISAT2 version 2.2.1 [41] including parameters --known-splicesite-infile to take splice sites based on IBSC v2.41 annotation into account as well as --rna-strandness for strand-specific information. The aligned reads were filtered to eliminate non proper-paired reads, and low quality (< 30) alignments, and then sorted using SAMtools version 1.6 [42]. The mapping procedure was repeated using newer version of the reference genome, Morex v3 (Ensembl Plants server; https://plants.ensembl.org/Hordeum_vulgare) [17].

### Differential gene expression analysis

For analyzing the differentially expressed transcripts (DET), counts of the mapped proper-paired reads were extracted by featureCounts from Subread package [43] in R software [39] based on annotations of the Morex reference genome. Transcripts with expression levels greater than three counts in three libraries were retained. Normalization and differential gene expression analysis was performed with the R/Bioconductor package “edgeR” version 3.36.0 [44] in R software. Normalization was performed within the library according to Trimmed Mean of M-values (TMM) as well as reads per kilobase per million (RPKM). Hierarchical cluster analysis between individual libraries was done in R software in order to verify the quality of biological replication and only transcripts with expression levels ≥ 0.9 log_2_RPKM in at least two libraries for each tissue were retained. DETs were called at a false discovery rate (FDR) < 0.00001. Additionally, DETs detected between individual developmental stages at least in one tissue required both FDR < 0.00001 and an absolute log_2_fold-change > 2 in at least either of time-points 11 or 19 DAG, in order to be included in further analysis. Furthermore, DET not significantly expressed in the time-point 11 DAG in both apex and leaf tissues were eliminated. The heatmap was generated using package “pheatmap” version 1.0.12 [45] in R software. To get an overview of the SNP or InDel variants present in the gene transcripts, the legacy algorithm of Freebayes v1.3.6 [46] was used to call variants with a minimum fraction of alternate allele observations of 90%, a minimum alternate allele count of 2, a minimum coverage of 4, and minimum base and mapping qualities of 30. Then the output was filtered using BCFtools v1.17 [47] with the parameters of QUAL > = 30, and FORMAT/DP > = 14. The SNPs and InDels that appeared in only one sample or were present in samples of both genotypes were removed. The intersect function from BEDTools v2.30.0 [48] was used to identify overlaps with gene models using the GTF file of IBSC v2.41 annotation [15].

### Sequence analysis of the candidate gene

The full sequence of the candidate gene *HvSpt6* including promoter region was generated by amplification of DNA from DH lines using Sanger sequencing. The alignment of sequenced regions was performed by MegAlign Pro (Lasergene 7.1: DNASTAR Inc., Madison, WI). Identification of DNA binding motifs within the promoter was performed by Mulan [46] and binding sites were validated using the Universal Protein Knowledgebase (https://www.uniprot.org). To explore the natural variation of *HORVU1Hr1G067820* gene sequence in other barley accessions, IPK Galaxy Blast Suite (https://galaxy-web.ipk-gatersleben.de/) was used to BLAST the gene coding sequence to the Nucleotide BLAST database available in this website. The sequence of the coding region of the gene including exons and introns was downloaded for eight diverse accessions including European originated accessions Morex, Hockett, Golden Promise, Igri, and Barke, as well as East Asian originated accessions Akashinriki, ZDM02064, and ZDM01467. The sequence of the genes were aligned against coding sequence of *HORVU1Hr1G067820*, and phylogenetic analysis was performed based on Maximum likelihood method using MEGA v11.0.13 [47].

## Results

### Genetic recombinations in *HvHeading* QTL interval

Genotyping the *HvHeading* interval with 12 SNPs revealed no genetic recombination events in 534 MAGIC DH lines. Further genotyping of additional 279 DH lines in the interval using the most associated SNP marker at 71.03 cM (BOPA1_1016-376) and three adjacent markers SCRI_RS_138527 (70.40 cM), BOPA1_6655-978 (70.89 cM) and SCRI_RS_181239 (71.17 cM) also revealed no recombination events. This suggested that *HvHeading* interval might be a region with low possibility of recombination.

**Table 1:** Genotyping of *Ppd-H1*, *Vrn-H1* and *Vrn-H3* genes in parental lines of spring barley MAGIC population

Parents	*Ppd-H1*										*Vrn-H3*			*Vrn-H1*	
	SNP4	SNP5	SNP6	SNP7	SNP10	SNP 12	SNP14	SNP 15	SNP16	Allele	SNP 270	SNP 384	Allele	Deletion in intron1	Allele
Ack. Bavaria	C	C	C	C	C	C	G	T	G	*ppd-H1*	T	C	*vrn-H3*	Del	*Vrn-H1*
Ack. Danubia	C	C	C	C	C	C	G	T	G	*ppd-H1*	T	C	*vrn-H3*	Del	*Vrn-H1*
Criewener403	C	C	C	C	C	C	G	T	G	*ppd-H1*	T	C	*vrn-H3*	Del	*Vrn-H1*
Heils Franken	C	C	C	C	C	C	G	T	G	*ppd-H1*	T	C	*vrn-H3*	Del	*Vrn-H1*
Heines Hanna	C	C	C	C	C	C	G	T	G	*ppd-H1*	T	C	*vrn-H3*	Del	*Vrn-H1*
Pflugs Intensiv	C	C	C	C	C	C	G	T	G	*ppd-H1*	T	C	*vrn-H3*	Del	*Vrn-H1*
Barke	C	C	C	C	C	C	G	T	G	*ppd-H1*	T	C	*vrn-H3*	Del	*Vrn-H1*
Ragusa	G	T	T	G	T	A	A	C	A	*Ppd-H1*	A	G	*Vrn-H3*	_	*vrn-H1*
Reference	[6]										[7]			[37]	

### Selecting the contrasting pairs of DH lines and microscopy-based phenotyping of main shoot apex development

**Fig. 1:**
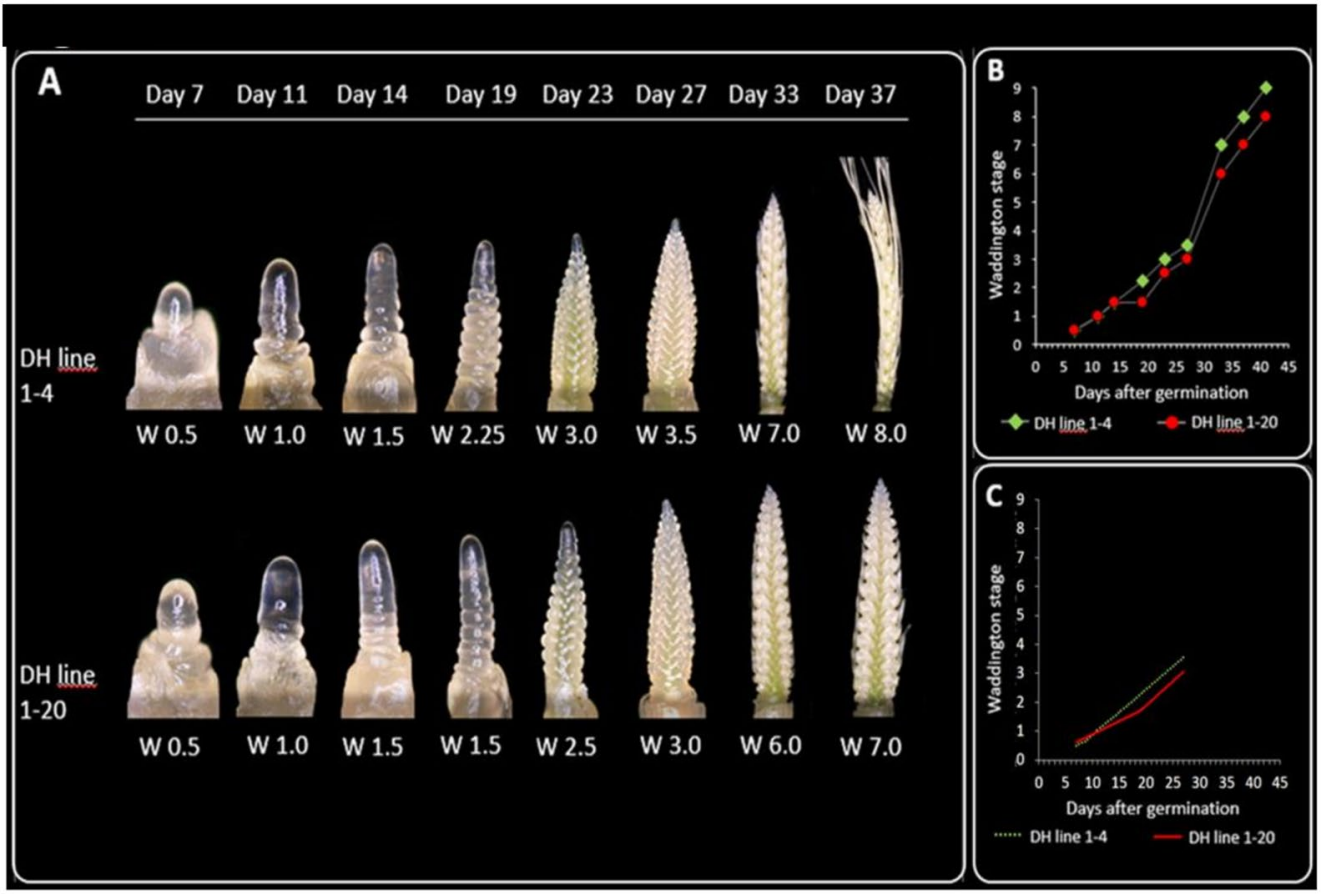
Comparing development of main shoot apical meristems in MAGIC DH lines 1-4 vs 1-20. **A**) Phenotypes of apex development under LD from 7 to 37 days after germination. **B**) Plot describing the apex development under LD from 7 to 37 days after germination. **C**) Broken-line regression analysis showed a statistically significant change of the slope for apex development of DH line 1-20 under LD from 7 to 27 days after germination

The *HvHeading* QTL was reported to be involved in prominent epistatic interactions with *Ppd-H1*, *Vrn-H1*, and *Vrn-H3* loci and these four loci explained a large portion of phenotypic variance for days to heading in spring barley MAGIC DH lines [5]. Our hypothesis was to screen MAGIC DH lines using genetic variations already described for *Ppd-H1*, *Vrn-H1*, and *Vrn-H3* genes to find the DH lines with the same alleles in loci with epistatic interaction to *HvHeading*. These selected lines would then serve as contrasting DH lines to phenotype the reported delayed-flowering phenotypic effect. The genotyping results of Sanger sequencing for nine SNPs for *Ppd-H1* gene [6], two SNPs for *Vrn-H3* [7], and a deletion in intron 1 of *Vrn-H1* [37], showed that only parental line Ragusa carried a different allele for all three genes (Table 1). After screening the MAGIC DH lines to find the contrasting pair, DH lines 1-4 and 1-20 were selected, which both carry parental haplotypes that harbor photoperiod-sensitive *Ppd-H1*, recessive *vrn-H3,* and winter-type *Vrn-H1* allele (Table S3-6). Furthermore, haplotypes in the *HvHeading* interval for DH lines 1-20 and 1-4 corresponded to Danubia and Barke parental lines respectively. The main shoot of line 1-20 headed five days (49 DAG) later than 1-4 (44 DAG). Microscopy-based phenotyping was performed to investigate the development of apex and inflorescence. Evaluation of apex development stages using paired Student’s t-test showed differences between DH lines. Line 1-20 was at early double-ridge stage (W1.5) on 19 DAG and showed slower development compared to line 1-4 (Fig. 1 A, B). From 23 to 27 DAG the development of both 1-4 and 1-20 moderately accelerated. At 27 DAG, 1-4 and 1-20 were at stamen primordium stage (W3.5) and lemma-floret primordium stage (W3.0) respectively. Their development sped up sharply from 27 to 33 DAG and continued with slower pace from 33 to 37 DAG. DH line 1–20 was constantly delayed compared to 1–4. Broken-line regression analysis revealed biphasic pattern for shoot apex development of 1–20 line and showed significant change of the slope under LD from 7 to 27 DAG. The slope of regression line from 7 to 19 DAG for 1–4 and 1–20 DH lines was 0.15 and 0.09, respectively, showing thus faster development rate for 1–4; the slope was similar for both from 23 to 27 DAG, 0.34 for 1–4 and 0.30 for 1–20 (Fig. 1C).

### Characterization of differential transcript expression for *HvHeading* in shoot apex and leaves

**Fig. 2:**
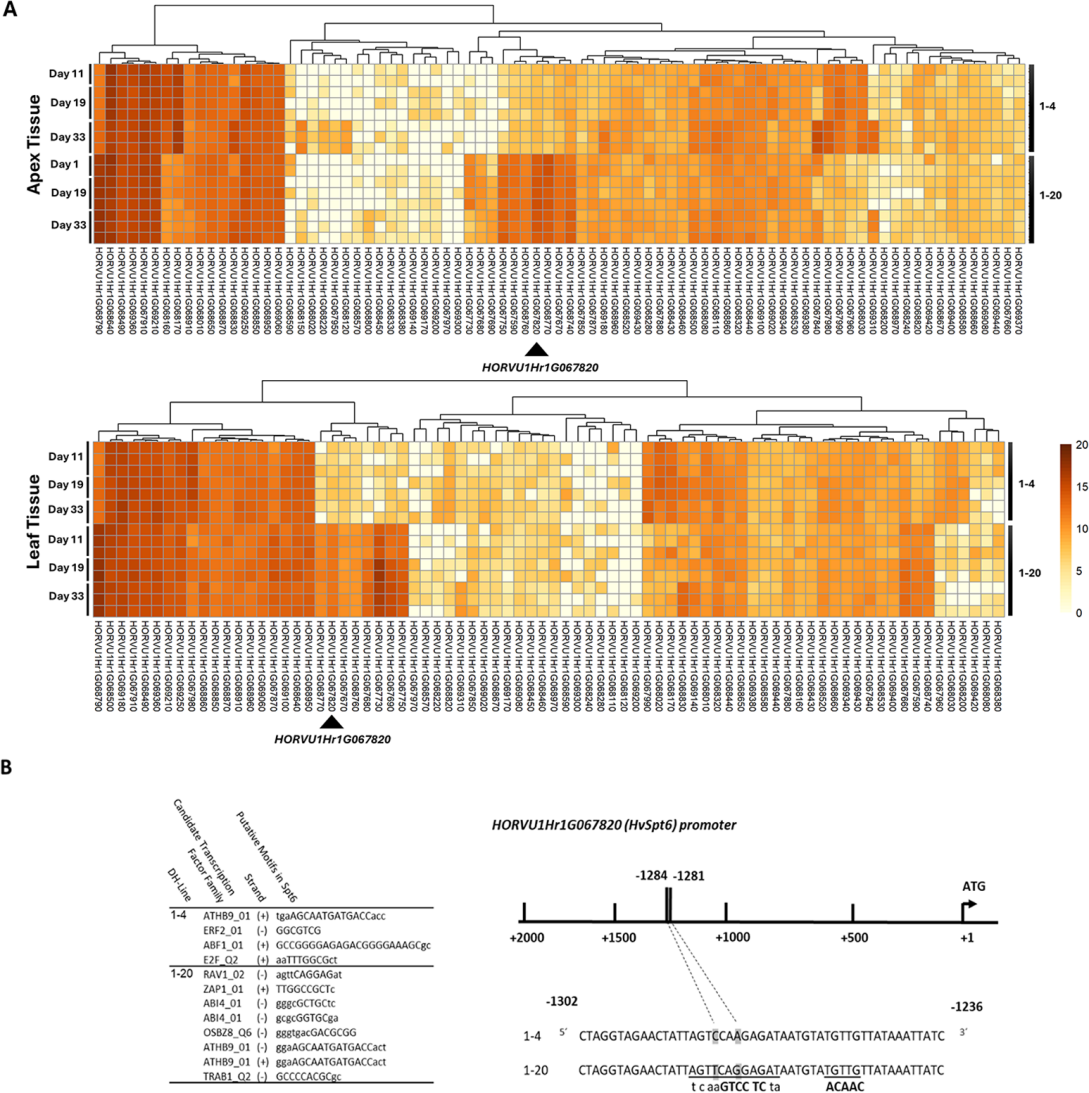
Overview of genes in *HvHeading* QTL interval. **A**) Heatmap of transcription expression in *HvHeading* QTL interval in apex and leaf tissue based on RNA-seq data normalized using RPKM approach for DH lines 1-4 and 1-20 at three time-points; 11, 19 and 33 days after germination. For each tissue, samples for each time-point are shown to be grouped together and there is a clear variation in gene expression according to time-points for each DH line. Higher expression is indicated by darker color. The black arrows show the position of the candidate gene with highly significant differential expression. **B**) Sequencing analysis of the promoter of *HORVU1Hr1G067820* showing the detected putative transcription factor binding sites specific to each DH-line (left) as well as the position of the putative TEM1 transcription factor binding site on promoter region of *HvSpt6* in DH line 1-20 (right)

RNA-seq analysis based on alignment of reads to Morex IBSC v2 reference genome, revealed quantitative differences in transcript expression for DH lines 1-4 and 1-20 at three time-points in apex and leaf tissues. Hierarchical cluster analysis clearly distinguished and grouped biological replicates of each time-point and genotype for apex tissue (Fig. 2 A). However, for apex tissue, one biological replicate from time-point 11 days for each DH line, and for leaf tissue one biological replicate for each DH line from time-point 19 days and one for DH line 1-4 in time point 33 days did not cluster with other biological replicates and were not included in downstream analysis. Results revealed differentially expressed transcripts for all time-points in apex and leaf tissues for both lines in *HvHeading* region (Table S7-8). The analysis showed total 22 differentially expressed genes in both tissues within an interval of 3.94 Mbs, from which 20 were shared (Table S9). The region was located inside the interval detected by Afsharyan et al. [5]. Gene ontology (GO) analysis for 22 genes in the interval, revealed overrepresentation of various GO classifications including biological processes such as regulation of DNA-templated transcription (regulation of DNA-templated transcription elongation (GO:0032784), regulation of DNA-templated transcription (GO:0006355)), and molecular functions such as nucleic acid binding (DNA binding (GO:0003677), DNA-binding transcription factor activity (GO:0003700), nucleic acid binding (GO:0003676), and sequence-specific DNA binding (GO:0043565)). Since phenotyping showed delayed development of shoot apex in line 1-20 compared to 1-4 On 19 DAG, therefore we hypothesized that the causative gene would show a change in expression in either or both 11 DAG and 19 DAG. Among the differentially expressed genes, genes such as *HORVU1Hr1G067590*,* HORVU1Hr1G067820*, and *HORVU1Hr1G067960* had higher fold-change for either or both 11 and 19 DAG compared to 33 DAG time-point in apex tissue. *HORVU1Hr1G067820* (*HORVU.MOREX.r3.1HG0069170* in Morex v3 reference genome) was the strongest differentially expressed gene for 11 and 19 DAG in apex tissue, and showed higher fold-change in these time-points compared to 33 DAG (11 DAG: 6.04 log_2_-fold-changes, 19 DAG: 6.39 log_2_-fold-changes, 33 DAG: 5.98 log_2_-fold changes) in apex tissue. This gene was annotated as a transcription elongation factor (*HvSpt6*) with associated GO terms, nucleic acid binding (GO:0003676), DNA binding (GO:0003677), nucleobase-containing compound metabolic process (GO:0006139), regulation of transcription by RNA polymerase II (GO:0006357) and regulation of DNA-templated transcription elongation (GO:0032784), and was up-regulated in DH line 1-20 compared to DH line 1-4 (Fig. 2 A, Table S9). Within the QTL interval, variant calling identified 77 SNP or InDel variants in the gene transcripts expressed in samples from the two genotypes (Table S10).

### Sequence analysis of conserved region and promoter analysis for the putative candidate gene *HORVU1Hr1G067820 (HvSpt6)*

Sanger sequencing of *HORVU1Hr1G067820* (*HvSpt6*) gene was performed for DH lines 1–4 and 1–20 to investigate the genetic variations. The sequence comparison revealed mutations across different putative transcription factor binding sites in the promoter region between the two lines including a mutation that created binding site for transcription factor *TEM1* in 1–20 (Fig. 2B). *TEM1* is a RAV family transcription factor which have been shown to bind bipartite DNA sequences [48, 49]. The gene coding sequence was identical in both DH lines 1–4 and 1–20. The genetic variation for *HORVU1Hr1G067820* gene was explored in eight accessions including European originated accessions Morex, Hockett, Golden Promise, Igri, and Barke, as well as East Asian originated accessions Akashinriki, ZDM02064, and ZDM01467. The results showed variations among accessions in exons and introns. Phylogenetic analysis using maximum likelihood method revealed that the East Asian accessions Akashinriki and ZDM02064 clustered together in a distinct subclade. In contrast, the other East Asian accession, ZDM01467, exhibited more similarity to European accessions.

## Discussion

### Background effect elimination facilitates candidate gene identification at *HvHeading* QTL

Identifying the gene underlying *HvHeading* QTL required removing the background effects that could mask the effect of *HvHeading.* The conventional approach involves developing near-isogenic lines (NILs) or mutations, which is a time-consuming and resource-intensive process [21, 25, 29]. This QTL exhibits a significant epistatic interaction with regions corresponding to major flowering time genes *Ppd-H1*, *Vrn-H1* and *Vrn-H3* [5]. Our approach focuses on selecting DH lines from the same population used for QTL mapping that carry the same alleles for the loci that are detected to have strong epistatic interaction with *HvHeading* for controlling flowering time in spring barley MAGIC population. These loci are presumed to control a high proportion of the trait phenotypic variation in this population [5]. This approach relies on precise mapping of QTL associated with the trait of interest as well as availability of homozygote lines from a high resolution mapping population. Efficiency in detecting trait-specific-near-isogenic regions also depends on complexity of trait. When the trait is controlled by many small effect QTL, screening for DH lines that carry the desired allele combination and create a distinguishing phenotypic effect becomes more challenging. The DH line 1–4 and 1–20 carried the same alleles for *Ppd-H1*, *Vrn-H1* and *vrn-H3* and line 1–20 which carried the allele descending from Danubia in *Hvheading* interval showed a delayed apex and inflorescence development compared to DH line 1–4. Therefore, the strategy to use mapped epistatic flowering time regions for eliminating the background effect of other important flowering time genes resulted in finding MAGIC DH lines which successfully revealed the expected flowering-delaying effect of *HvHeading* QTL allele.

### Prioritization of genes in *HvHeading* interval and detecting *HvSpt6* as a candidate gene

The *HvHeading* QTL is located on the long arm of chromosome 1H. The genotyping of *HvHeading* interval using 813 MAGIC DH lines found no recombination in this region. Gene identification in suppressed recombination regions of barley genome is a tedious process since targeting these regions by performing traditional approaches such as fine mapping is less effective [26, 50]. RNA-seq approach was performed to provide a validation for differential gene expression in *HvHeading* region among contrasting pair of DH lines for a candidate gene approach. Even though, differential gene expression approach is not the only criteria for identifying candidate genes, it provides a link between gene activity and the phenotype, making it a targeted approach to identify candidate genes involved in similar biological processes. This is in contrast to other approaches such as variant calling, which identifies genetic variants which require further functional validation to determine if they are linked with gene function. *HvHeading* is expected to cause delaying effect on apex development, which is demonstrated from double-ridge stage in main shoot apex of DH line 1–20. This suggests that the expression of the gene underling this QTL is eventually expected before or during double-ridge stage which includes first and second time-points. RNA analysis using Morex IBSC v2 [15] as reference genome revealed several differentially expressed genes within the QTL interval, including those related to DNA and transcript binding functions. Finding several differentially expressed genes linked with similar biological processes strengthens the reliability of candidate gene selection within this region. A transcription elongation factor, *HORVU1Hr1G067820* gene, which is potentially a homolog of Arabidopsis *Spt6* gene showed different expression patterns among DH line 1–4 and 1–20 consistent with the observed *HvHeading* phenotype in apex. The analysis identified a complex situation regarding gene models across different reference genome versions. In Morex IBSC v2 reference genome, we identified another gene, *HORVU0Hr1G001120*, positioned on an unlocated chromosome, which showed high sequence similarity to *HORVU1Hr1G067820* (*HvSpt6)*. Both genes correspond to a single gene model, *HORVU.MOREX.r3.1HG0069170*, in the Morex v3 reference genome [17], located on chromosome 1H. This suggests that initial positioning of *HORVU0Hr1G001120* was likely due to a misassembly in the IBSC v2 genome, and the corrected location on 1H chromosome is represented in the Morex v3 reference genome. When RNA-seq reads from *HORVU1Hr1G067820* and *HORVU0Hr1G001120* were combined for differential gene expression analysis using Morex IBSC v2, the results showed statistical significance, despite the fold change being below 2 (Table S11). This, along with the reports that homologous genes play a role in flowering time in other cereals such as rice, supports *HORVU1Hr1G067820* as a possible candidate gene for the *HvHeading* QTL.

When repeating the RNA-seq analysis with the Morex v3 reference genome, the anticipated differential gene expression between genotypes was not observed (Tables S12–S13). This discrepancy highlights substantial limitations in relying solely on differential gene expression analysis across different reference genome versions, and suggests that the evidence for *HvSpt6* as the candidate gene underlying *HvHeading* QTL should be considered preliminary. Additional complementary approaches, including validation of gene expression using qRT-PCR and functional validation of the candidate gene, might be necessary to conclusively establish the candidate gene associated with *HvHeading*.

Further investigation into the natural variation of this gene in eight barley accessions revealed a distinction between two of the East Asian originated accessions in a subclade from European ones. This finding suggests that *HvSpt6* gene was possibly involved in diversification of barley. It is reported that *Spt6* gene is involved in *Histone H3 lysine 36* (*H3K36*) methylation that mediates epigenetic regulation of flowering [51] and affects temperature-induced alternative splicing and flowering in plants [52]. *H3K36* methylation is also reported to have a critical role in timing of flowering in rice [53]. *Spt6* gene is reported to code a conserved transcription elongation factor, that controls transcription and chromatin structure and transcript initiation [54–58]. The role of histone and chromatin modifiers have been extensively studied in flowering time pathway of *Arabidopsis* [59, 60]. Analysis of promoter region revealed several genetic variations in DH line 1–20 including two mutations that created a binding cite for *TEM1* transcription factor. *TEM1* (with the combinatorial recognition consensus motif C(A/C/G)ACA(N)2–8(C/A/T)ACCTG) belongs to *RAV* family of transcription factors [48] and is known to suppress flowering in Arabidopsis by deposition of *H3K27me3* at the 5′-UTR of *FT* gene [49]. Considering the reports for involvement of *Spt6* in histone methylation, which plays a critical role in flowering time in plants, particularly cereals, this gene could be considered as the candidate gene underlying *HvHeading*.

While our analyses and the existing literature support *HvSpt6* as one possible candidate gene that might be involved in the switch from vegetative to reproductive stage, substantial additional evidence would be required to confirm this hypothesis. Additional studies are required to validate its expression using qRT-PCR and clarify its functional role within the flowering time regulatory network of barley. Moreover, potential inconsistencies across different barley reference genome versions underscore the need for caution and highlight the importance of integrating multiple genomic resources to strengthen future conclusions.

## Conclusions

This study presents results validating the phenotyping effect of *HvHeading* locus as a flowering time QTL; and then utilizes comparative transcriptomics to prioritize candidate genes by identifying differentially expressed genes in the interval. This approach does not require developing a fine-mapping population and therefore is useful when analyzing genomes with extensive regions of suppressed recombination. We identified a transcription elongation factor *HvSpt6* gene as a possible candidate gene for *HvHeading*. Further studies are needed to validate and functionally characterize the candidate gene and investigate its role in flowering time of barley. Our study suggests that *HvHeading* can be targeted to fine-tune flowering time in barley as an important breeding trait to further improve crop yield in barley and related crops species.

## Electronic supplementary material

Below is the link to the electronic supplementary material.

**Supplementary Material 1**: **Table S1**. The list of SNP markers in the *HvHeading* QTL interval according to barley 9k iSelect array, and the corresponding positions in reference genomes Morex IBSC v2 and Morex v3. **Table S2**. Primers description for genotyping of *Ppd-H1*, *Vrn-H1* and *Vrn-H3* in parental lines of spring barley MAGIC population. **Table S3**. Genotyping of *Ppd-H1* locus using SNP markers for founders of spring barley MAGIC population and spring barley MAGIC DH lines 1–4 and 1–20. **Table S4**. Genotyping of *Vrn-H1* locus using SNP markers for founders of spring barley MAGIC population and spring barley MAGIC DH lines 1–4 and 1–20. **Table S5**. Genotyping of *Vrn-H3* locus using SNP markers for founders of spring barley MAGIC population and spring barley MAGIC DH lines 1–4 and 1–20. **Table S6**. Parental haplotype blocks for loci harboring *HvHeading* interval, *Ppd-H1*, *Vrn-H1* and *Vrn-H3* genes in spring barley MAGIC DH lines 1–4 and 1–20, and the allele corresponding to each gene. **Table S7**. Transcription expression in *HvHeading* QTL interval in apex tissue based on alignment of RNA-seq data to Morex IBSC v2 reference genome, and normalized using RPKM approach for DH lines 1–4 and 1–20 in three time-points; 11, 19, and 33 days after germination. **Table S8**. Transcription expression in *HvHeading* QTL interval in leaf tissue based on alignment of RNA-seq data to Morex IBSC v2 reference genome, and normalized using RPKM approach for DH lines 1–4 and 1–20 in three time-points 11, 19, and 33 days after germination. **Table S9**. Differentially expressed genes in the *HvHeading* interval (1 H chromosome) based on Morex IBSC v2 reference genome comparing barley MAGIC DH-line 1–20 to DH-line 1–4 for three time-points 11, 19, and 33 days after sowing (DAS). **Table S10**. Overview of the SNPs and InDels found in the expressed transcripts within the *HvHeading* interval (1 H chromosome) in barley MAGIC DH-line 1–20 to DH-line 1–4 including all tissues and time-points. **Table S11**. Differential gene expression analysis for *HORVU1Hr1G067820*, *HORVU0Hr1G001120* and their cumulative reads based on alignment to Morex IBSC v2 reference genome, comparing barley MAGIC DH-line 1–20 to DH-line 1–4 for three time-points 11, 19, and 33 days after sowing (DAS). **Table S12**. Transcription expression in *HvHeading* QTL interval in apex tissue based on alignment of RNA-seq data to Morex v3 reference genome, and normalized using RPKM approach for DH lines 1–4 and 1–20 in three time-points; 11, 19, and 33 days after germination. **Table S13**. Transcription expression in *HvHeading* QTL interval in leaf tissue based on alignment of RNA-seq data to Morex v3 reference genome, and normalized using RPKM approach for DH lines 1–4 and 1–20 in three time-points; 11, 19, and 33 days after germination.

## Data Availability

All raw data generated in this study including RNA sequencing data have been submitted to the NCBI BioProject database (https://www.ncbi.nlm.nih.gov/bioproject/) under accession number PRJNA1182550.

## References

[1] Harlan JR, Zohary D. Distribution of wild wheats and Barley - The present distribution of wild forms May provide clues to the regions of early cereal domestication. Sci (80-). 1966;153:1074–80. 10.1126/science.153.3740.1074 17737582

[2] Ross-Ibarra J, Morrell PL, Gaut BS. Plant domestication, a unique opportunity to identify the genetic basis of adaptation. Proc Natl Acad Sci. 2007;104 Supplement 1:8641–8. 10.1073/pnas.0700643104 PMC1876441 17494757

[3] Cockram J, Jones H, Leigh FJ, O’Sullivan D, Powell W, Laurie DA, et al. Control of flowering time in temperate cereals: genes, domestication, and sustainable productivity. J Exp Bot. 2007;58:1231–44. 17420173 10.1093/jxb/erm042

[4] Maurer A, Draba V, Jiang Y, Schnaithmann F, Sharma R, Schumann E, et al. Modelling the genetic architecture of flowering time control in barley through nested association mapping. BMC Genomics. 2015;16:1–12. 25887319 10.1186/s12864-015-1459-7 PMC4426605

[5] Afsharyan NP, Sannemann W, Léon J, Ballvora A. Effect of epistasis and environment on flowering time in barley reveals a novel flowering-delaying QTL allele. J Exp Bot. 2020;71:893–906. 31781747 10.1093/jxb/erz477 PMC6977191

[6] Turner A, Beales J, Faure S, Dunford RP, Laurie DA. Botany: the pseudo-response regulator Ppd-H1 provides adaptation to photoperiod in barley. Sci (80-). 2005;310:1031–4. 10.1126/science.1117619 16284181

[7] Yan L, Fu D, Li C, Blechl A, Tranquilli G, Bonafede M, et al. The wheat and barley vernalization gene VRN3 is an orthologue of FT. Proc Natl Acad Sci. 2006;103:19581–6. 17158798 10.1073/pnas.0607142103 PMC1748268

[8] Distelfeld A, Li C, Dubcovsky J. Regulation of flowering in temperate cereals. Curr Opin Plant Biol. 2009;12:178–84. 19195924 10.1016/j.pbi.2008.12.010

[9] Hemming MN, Fieg S, Peacock WJ, Dennis ES, Trevaskis B. Regions associated with repression of the barley (Hordeum vulgare) VERNALIZATION1 gene are not required for cold induction. Mol Genet Genomics. 2009;282:107–17. 19404679 10.1007/s00438-009-0449-3

[10] Rollins JA, Drosse B, Mulki MA, Grando S, Baum M, Singh M, et al. Variation at the vernalisation genes Vrn-H1 and Vrn-H2 determines growth and yield stability in barley (Hordeum vulgare) grown under dryland conditions in Syria. Theor Appl Genet. 2013;126:2803–24. 23918065 10.1007/s00122-013-2173-y

[11] Künzel G, Korzun L, Meister A. Cytologically integrated physical restriction fragment length polymorphism maps for the barley genome based on translocation breakpoints. Genetics. 2000;154:397–412. 10628998 10.1093/genetics/154.1.397 PMC1460903

[12] Schneeberger K, Ossowski S, Lanz C, Juul T, Petersen AH, Nielsen KL, et al. SHOREmap: simultaneous mapping and mutation identification by deep sequencing. Nat Methods. 2009;6:550–1. 19644454 10.1038/nmeth0809-550

[13] Takagi H, Tamiru M, Abe A, Yoshida K, Uemura A, Yaegashi H, et al. MutMap accelerates breeding of a salt-tolerant rice cultivar. Nat Biotechnol. 2015;33:445. 25798936 10.1038/nbt.3188

[14] Mayer KFX, Waugh R, Langridge P, Close TJ, Wise RP, Graner A, et al. A physical, genetic and functional sequence assembly of the barley genome. Nature. 2012;491:711–6. 23075845 10.1038/nature11543

[15] Mascher M, Gundlach H, Himmelbach A, Beier S, Twardziok SO, Wicker T, et al. A chromosome conformation capture ordered sequence of the barley genome. Nature. 2017;544:427–33. 28447635 10.1038/nature22043

[16] Monat C, Padmarasu S, Lux T, Wicker T, Gundlach H, Himmelbach A, et al. TRITEX: Chromosome-scale sequence assembly of triticeae genomes with open-source tools. Genome Biol. 2019;20:1–18. 31849336 10.1186/s13059-019-1899-5 PMC6918601

[17] Mascher M, Wicker T, Jenkins J, Plott C, Lux T, Koh CS, et al. Long-read sequence assembly: A technical evaluation in barley. Plant Cell. 2021;33:1888–906. 33710295 10.1093/plcell/koab077 PMC8290290

[18] Monclus R, Leplé J-C, Bastien C, Bert P-F, Villar M, Marron N, et al. Integrating genome annotation and QTL position to identify candidate genes for productivity, architecture and water-use efficiency in Populus spp. BMC Plant Biol. 2012;12:173. 23013168 10.1186/1471-2229-12-173 PMC3520807

[19] Bargsten JW, Nap J-P, Sanchez-Perez GF, van Dijk ADJ. Prioritization of candidate genes in QTL regions based on associations between traits and biological processes. BMC Plant Biol. 2014;14:330. 25492368 10.1186/s12870-014-0330-3 PMC4274756

[20] Correa J, Mamani M, Muñoz-Espinoza C, Laborie D, Muñoz C, Pinto M, et al. Heritability and identification of QTLs and underlying candidate genes associated with the architecture of the grapevine cluster (Vitis vinifera L). Theor Appl Genet. 2014;127:1143–62. 24556794 10.1007/s00122-014-2286-y

[21] Schneeberger K. Using next-generation sequencing to isolate mutant genes from forward genetic screens. Nat Rev Genet. 2014;15:662–76. 25139187 10.1038/nrg3745

[22] Beier S, Himmelbach A, Colmsee C, Zhang X-Q, Barrero RA, Zhang Q, et al. Construction of a map-based reference genome sequence for barley, Hordeum vulgare L. Sci Data. 2017;4:170044. 28448065 10.1038/sdata.2017.44 PMC5407242

[23] van Esse GW, Walla A, Finke A, Koornneef M, Pecinka A, von Korff M. Six-Rowed Spike3 (VRS3) is a histone demethylase that controls lateral spikelet development in barley. Plant Physiol. 2017;174:2397–408. 28655778 10.1104/pp.17.00108 PMC5543938

[24] Gudys K, Guzy-Wrobelska J, Janiak A, Dziurka M, Ostrowska A, Hura K, et al. Prioritization of candidate genes in QTL regions for physiological and biochemical traits underlying drought response in barley (Hordeum vulgare L). Front Plant Sci. 2018;9:769. 29946328 10.3389/fpls.2018.00769 PMC6005862

[25] Kumar J, Gupta D, Sen, Gupta S, Dubey S, Gupta P, Kumar S. Quantitative trait loci from identification to exploitation for crop improvement. Plant Cell Rep. 2017;36:1187–213. 28352970 10.1007/s00299-017-2127-y

[26] Sánchez-Martín J, Steuernagel B, Ghosh S, Herren G, Hurni S, Adamski N, et al. Rapid gene isolation in barley and wheat by mutant chromosome sequencing. Genome Biol. 2016;17:1–7. 27795210 10.1186/s13059-016-1082-1 PMC5087116

[27] Shmakov NA, Vasiliev GV, Shatskaya NV, Doroshkov AV, Gordeeva EI, Afonnikov DA et al. Identification of nuclear genes controlling chlorophyll synthesis in barley by RNA-seq. BMC Plant Biol. 2016;16 Suppl 3. 10.1186/s12870-016-0926-x PMC5123340 28105957

[28] Liller CB, Walla A, Boer MP, Hedley P, Macaulay M, Effgen S, et al. Fine mapping of a major QTL for Awn length in barley using a multiparent mapping population. Theor Appl Genet. 2017;130:269–81. 27734096 10.1007/s00122-016-2807-y PMC5263209

[29] Mascher M, Jost M, Kuon JE, Himmelbach A, Abfalg A, Beier S, et al. Mapping-by-sequencing accelerates forward genetics in barley. Genome Biol. 2014;15:1–15. 10.1186/gb-2014-15-6-r78 PMC4073093 24917130

[30] Cavanagh C, Morell M, Mackay I, Powell W. From mutations to MAGIC: resources for gene discovery, validation and delivery in crop plants. Curr Opin Plant Biol. 2008;11:215–21. 18295532 10.1016/j.pbi.2008.01.002

[31] King EG, Merkes CM, McNeil CL, Hoofer SR, Sen S, Broman KW, et al. Genetic dissection of a model complex trait using the Drosophila synthetic population resource. Genome Res. 2012;22:1558–66. 22496517 10.1101/gr.134031.111 PMC3409269

[32] Sannemann W, Huang BE, Mathew B, Léon J. Multi-parent advanced generation inter-cross in barley: high-resolution quantitative trait locus mapping for flowering time as a proof of concept. Mol Breed. 2015;35:86.

[33] Comadran J, Kilian B, Russell J, Ramsay L, Stein N, Ganal M, et al. Natural variation in a homolog of Antirrhinum CENTRORADIALIS contributed to spring growth habit and environmental adaptation in cultivated barley. Nat Genet. 2012;44:1388–91. 23160098 10.1038/ng.2447

[34] Cantalapiedra CP, Boudiar R, Casas AM, Igartua E, Contreras-Moreira B. BARLEYMAP: physical and genetic mapping of nucleotide sequences and annotation of surrounding loci in barley. Mol Breed. 2015;35:13.

[35] Colmsee C, Beier S, Himmelbach A, Schmutzer T, Stein N, Scholz U, et al. BARLEX - The barley draft genome explorer. Mol Plant. 2015;8:964–6. 25804976 10.1016/j.molp.2015.03.009

[36] Bolser D, Staines DM, Pritchard E, Kersey P. Ensembl plants: integrating tools for visualizing, mining, and analyzing plant genomics data. Plant bioinformatics. Springer; 2016. pp. 115–40. 10.1007/978-1-4939-3167-5_6 26519403

[37] Wang G, Schmalenbach I, von Korff M, Léon J, Kilian B, Rode J, et al. Association of barley photoperiod and vernalization genes with QTLs for flowering time and agronomic traits in a BC2DH population and a set of wild barley introgression lines. Theor Appl Genet. 2010;120:1559–74. 20155245 10.1007/s00122-010-1276-y PMC2859222

[38] Waddington SR, Cartwright PM, Wall PC. A quantitative scale of Spike initial and pistil development in barley and wheat. Ann Bot. 1983;51:119–30.

[39] Core Team R, Team A. RC. R: A language and environment for statistical computing. R Foundation for Statistical Computing, Vienna, Austria. 2012. 2022.

[40] Bolger AM, Lohse M, Usadel B. Trimmomatic: a flexible trimmer for illumina sequence data. Bioinformatics. 2014;30:2114–20. 24695404 10.1093/bioinformatics/btu170 PMC4103590

[41] Kim D, Langmead B, Salzberg SL. HISAT: a fast spliced aligner with low memory requirements. Nat Methods. 2015;12:357–60. 25751142 10.1038/nmeth.3317 PMC4655817

[42] Li H, Handsaker B, Wysoker A, Fennell T, Ruan J, Homer N, et al. The sequence alignment/map format and samtools. Bioinformatics. 2009;25:2078–9. 19505943 10.1093/bioinformatics/btp352 PMC2723002

[43] Liao Y, Smyth GK, Shi W. FeatureCounts: an efficient general purpose program for assigning sequence reads to genomic features. Bioinformatics. 2014;30:923–30. 24227677 10.1093/bioinformatics/btt656

[44] Robinson MD, McCarthy DJ, Smyth GK. EdgeR: a bioconductor package for differential expression analysis of digital gene expression data. Bioinformatics. 2010;26:139–40. 19910308 10.1093/bioinformatics/btp616 PMC2796818

[45] Kolde R, Kolde MR. Package ‘pheatmap’. R Packag. 2015;1:790.

[46] Garrison E, Marth G. Haplotype-based variant detection from short-read sequencing. 2012. arXiv preprint arXiv:1207.3907. 10.48550/arXiv.1207.3907.

[47] Danecek P, Bonfield JK, Liddle J, Marshall J, Ohan V, Pollard MO, et al. Twelve years of SAMtools and BCFtools. GigaScience. 2021;10:giab008. 10.1093/gigascience/giab0. 10.1093/gigascience/giab008 PMC7931819 33590861

[48] Quinlan AR, Hall IM. BEDTools: a flexible suite of utilities for comparing genomic features. Bioinformatics. 2010;26:841–2. 10.1093/bioinformatics/btq033. 10.1093/bioinformatics/btq033 PMC2832824 20110278

[49] Hu H, Tian S, Xie G, Liu R, Wang N, Li S, et al. TEM1 combinatorially binds to FLOWERING LOCUS T and recruits a polycomb factor to repress the floral transition in Arabidopsis. Proc Natl Acad Sci U S A. 2021;118:1–8. 10.1073/pnas.2103895118 PMC8536356 34446554

[50] Hatta MAM, Steuernagel B, Wulff BBH. Chapter 4 Rapid Gene Cloning in Wheat. 2019. pp. 65–95.

[51] Shi J, Dong A, Shen WH. Epigenetic regulation of rice flowering and reproduction. Front Plant Sci. 2015;5 JAN:1–13. 10.3389/fpls.2014.00803 PMC4309181 25674094

[52] Pajoro A, Severing E, Angenent GC, Immink RGH. Histone H3 lysine 36 methylation affects temperature-induced alternative splicing and flowering in plants. Genome Biol. 2017;18:1–12. 28566089 10.1186/s13059-017-1235-x PMC5452352

[53] Sui P, Shi J, Gao X, Shen WH, Dong A. H3K36 methylation is involved in promoting rice flowering. Mol Plant. 2013;6:975–7. 23239829 10.1093/mp/sss152

[54] Ardehali MB, Yao J, Adelman K, Fuda NJ, Petesch SJ, Webb WW, et al. Spt6 enhances the elongation rate of RNA polymerase II in vivo. EMBO J. 2009;28:1067–77. 19279664 10.1038/emboj.2009.56 PMC2683705

[55] Endoh M, Zhu W, Hasegawa J, Watanabe H, Kim D-K, Aida M, et al. Human Spt6 stimulates transcription elongation by RNA polymerase II in vitro. Mol Cell Biol. 2004;24:3324–36. 15060154 10.1128/MCB.24.8.3324-3336.2004 PMC381665

[56] Swanson MS, Carlson M, Winston F. SPT6, an essential gene that affects transcription in Saccharomyces cerevisiae, encodes a nuclear protein with an extremely acidic amino terminus. Mol Cell Biol. 1990;10:4935–41. 2201908 10.1128/mcb.10.9.4935-4941.1990 PMC361114

[57] Hartzog GA, Wada T, Handa H, Winston F. Evidence that Spt4, Spt5, and Spt6 control transcription elongation by RNA polymerase II InSaccharomyces cerevisiae. Genes Dev. 1998;12:357–69. 9450930 10.1101/gad.12.3.357 PMC316481

[58] Doris SM, Chuang J, Viktorovskaya O, Murawska M, Spatt D, Churchman LS, et al. Spt6 is required for the fidelity of promoter selection. Mol Cell. 2018;72:687–e6996. 30318445 10.1016/j.molcel.2018.09.005 PMC6239972

[59] Berr A, Shafiq S, Shen WH. Histone modifications in transcriptional activation during plant development. Biochim Biophys Acta - Gene Regul Mech. 2011;1809:567–76. 10.1016/j.bbagrm.2011.07.001 21777708

[60] Van Lijsebettens M, Grasser KD. Transcript elongation factors: shaping transcriptomes after transcript initiation. Trends Plant Sci. 2014;19:717–26. 25131948 10.1016/j.tplants.2014.07.002

